# Supplementary figures and images for: Thalamic, cortical, and amygdala involvement in the processing of a natural sound cue of danger
Source: PLoS Biol. 2020 May 12;18(5):e3000674. doi: 10.1371/journal.pbio.3000674 (PMC7217448; doi:10.1371/journal.pbio.3000674)

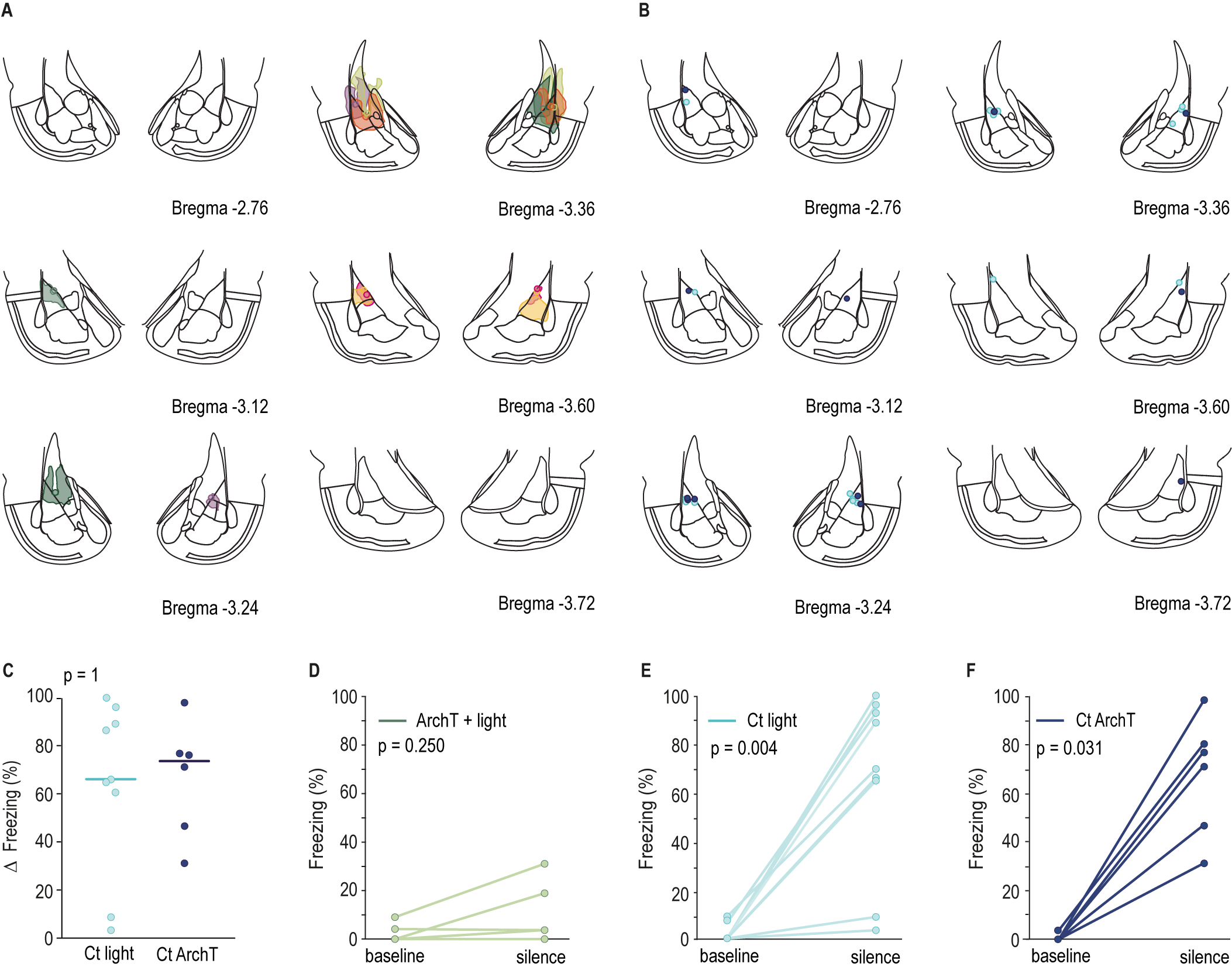

Supplement: S1 Fig — (A) Coronal slices representing fiber placements and spread of virus expression for ArchT + light group. (B) Fiber placement and/or injection site for the Ct light (light blue) and Ct ArchT (dark blue) groups. (C) Same as Fig 1D, but for animals of the 2 control groups. Horizontal bar represents the median value of the group (Ct light = 66.13%, Ct ArchT = 73.67%, Wilcoxon rank sum test, rank sum = 72) (S1 Data, Sheet S1 Fig). (D–F) Line graph showing average time spent freezing during the minute immediately preceding the cessation of the movement-evoked sound (baseline) and the minute of silence for each rat of the ArchT + light (n = 7), Ct light (n = 9), and Ct ArchT (n = 6) groups. Wilcoxon signed rank test. Baseline versus silence ArchT + light signedrank = 1; Ct light signedrank = 1; Ct ArchT signedrank = 0. (S1 Data, Sheet S1 Fig). ArchT, archaerhodopsin from Halorubrum strain TP009; Ct light, control light; Ct ArchT, control ArchT; LA, lateral amygdala. (TIF) [file pbio.3000674.s001.tif]

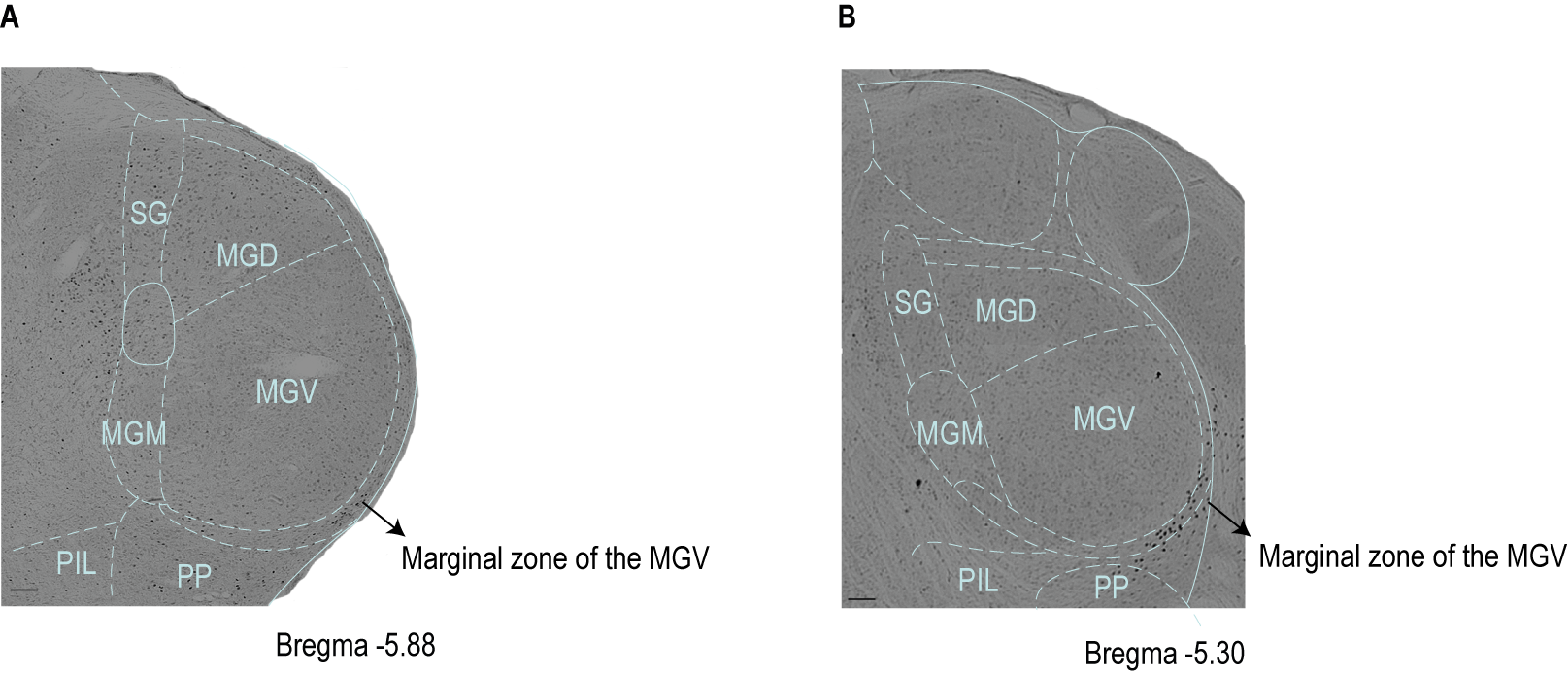

Supplement: S2 Fig — Representative images of coronal slices from rats exposed to silence gaps showing c-fos labeling in different subnuclei of the MGB. In particular, we observe c-fos-positive cells in the shell of the MGV, an area particularly responsive to the offset of sounds. c-fos-positive cells in this region were not quantified because it does not project directly to the LA. LA, lateral amygdala; MGB, medial geniculate body; MGV, ventral division of the MGB. (TIF) [file pbio.3000674.s002.tif]

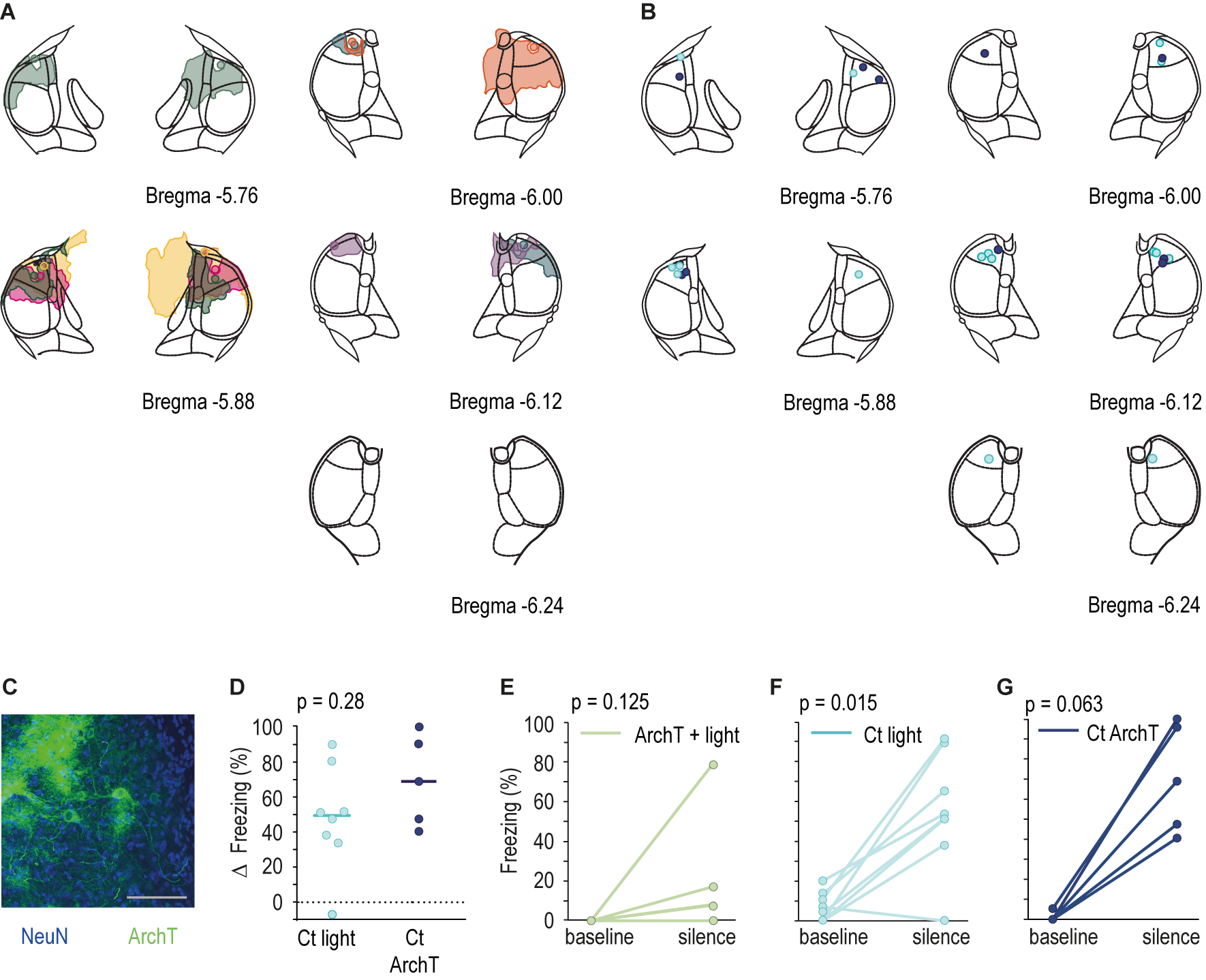

Supplement: S3 Fig — (A) Coronal slices representing fiber placements and spread of virus expression for ArchT + light group. (B) Fiber placement and/or injection site for the Ct light (light blue) and Ct ArchT (dark blue) groups. (C) Representative image of cells in MGD expressing ArchT-GFP. (D) Same as Fig 3D but for animals of the 2 control groups. Horizontal bar represents the median value (Ct light = 49.47%, Ct ArchT = 68.93%, Wilcoxon rank sum test, rank sum = 48) (S1 Data, Sheet S3 Fig). (E–G) Line graph showing average time spent freezing during the minute immediately preceding the cessation of the movement-evoked sound (baseline) and the minute of silence for each rat of the ArchT + light (n = 7), Ct light (n = 8), and Ct ArchT (n = 5) groups with surgeries targeting the MGD. Wilcoxon signed rank test, baseline versus silence ArchT + light signedrank = 0; Ct light signedrank = 1; Ct ArchT signedrank = 0. (S1 Data, Sheet S3 Fig). ArchT, archaerhodopsin from Halorubrum strain TP009; Ct light, control light; Ct ArchT, control Arch T; GFP, green fluorescent protein; LA, lateral amygdala; MGB, medial geniculate body; MGD, dorsal division of the MGB. (TIF) [file pbio.3000674.s003.tif]

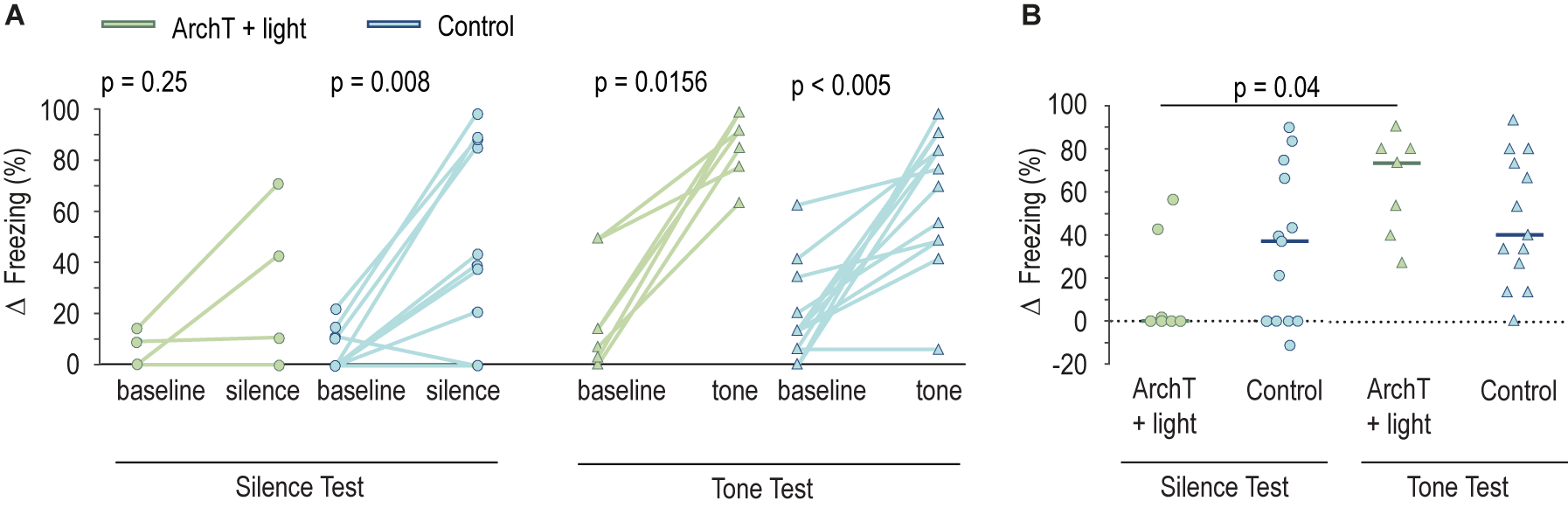

Supplement: S4 Fig — (A) Line graph showing average time spent freezing during the 15 seconds immediately preceding the cessation of the movement-evoked sound or tone (baseline) and the 15 seconds of stimulus (silence or tone) for each rat of the ArchT + light (n = 7) and Control (n = 13) groups. Wilcoxon signed rank test, baseline versus silence ArchT + light signedrank = 0; Control signedrank = 1; baseline versus tone ArchT + light signedrank = 0; Control signedrank = 0 (S1 Data, Sheet S4 Fig). (B) Individual dots represent the change in the percentage of time each animal spends freezing during the 15 seconds immediately preceding the cessation of the movement-evoked sound or tone (baseline) and the 15 seconds of stimulus (silence or tone) for each rat of the ArchT + light (n = 7) and Control (n = 13) groups. Horizontal bar represents the median value of the group (Silence test ArchT + light = 0.00%, Control = 37.07%; Tone test ArchT + light = 73.33%, Control = 40%). Kruskal–Wallis test chi-squared = 8 (S1 Data, Sheet S4 Fig) (S1 Data, Sheet S4 Fig). ArchT, archaerhodopsin from Halorubrum strain TP009; MGB, medial geniculate body; MGD, dorsal division of the MGB. (TIF) [file pbio.3000674.s004.tif]

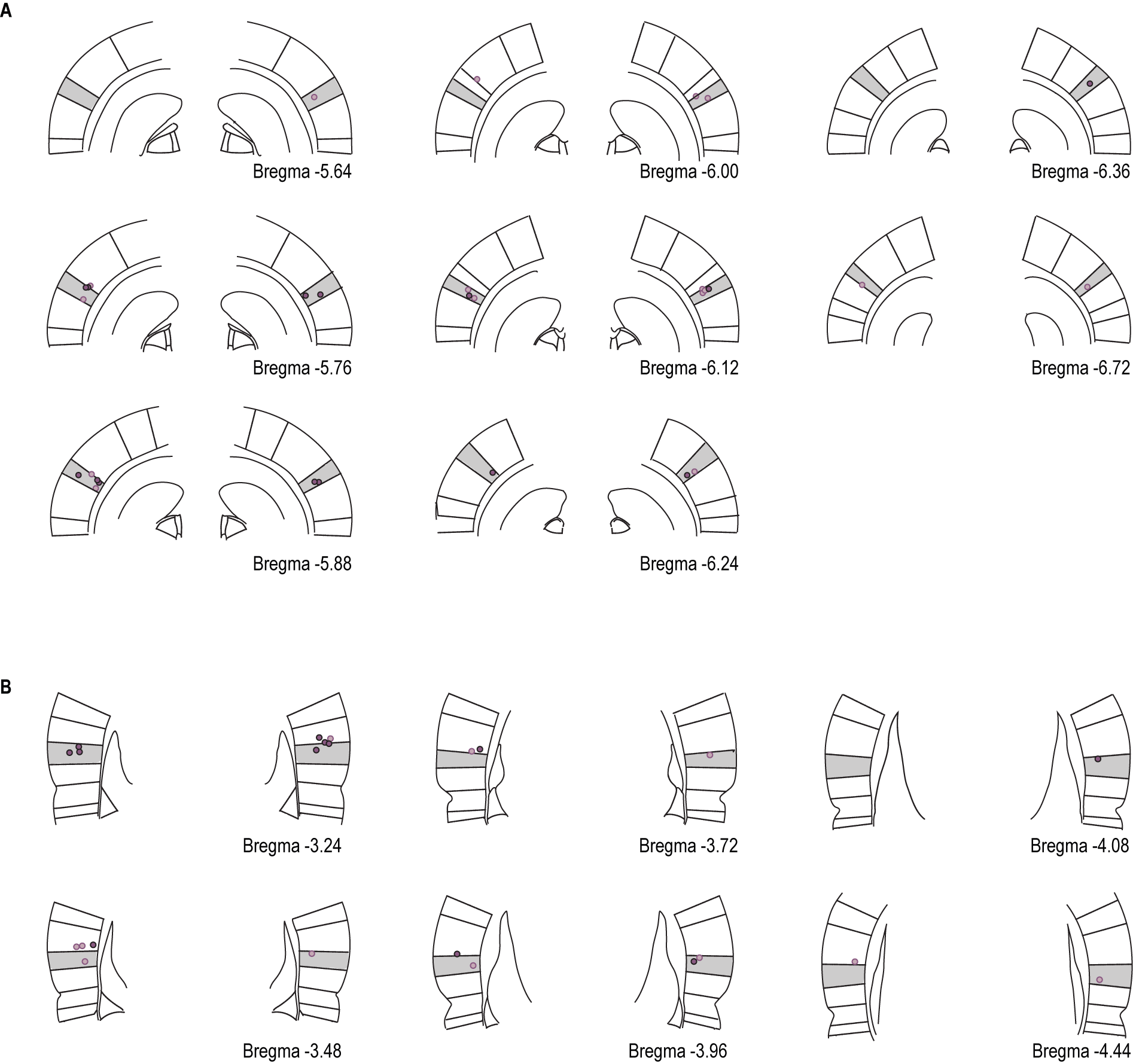

Supplement: S5 Fig — Coronal slices representing injection site for PBS (light purple) and muscimol (dark purple) in (A) PD and (B) VA. Areas shaded in gray correspond to PD and VA (respectively). PBS, phosphate buffered saline; PD, posterodorsal; VA, ventral area. (TIF) [file pbio.3000674.s005.tif]
