# Supplementary material for: Thalamic, cortical, and amygdala involvement in the processing of a natural sound cue of danger
Source: PLoS Biol. 2020 May 12;18(5):e3000674. doi: 10.1371/journal.pbio.3000674 (PMC7217448; doi:10.1371/journal.pbio.3000674)
Supplement: Attachment — Submitted filename: Pereira Response to Reviewer .pdf [file pbio.3000674.s008.pdf]

Please find below our point-by-point answer to all comments made by the reviewers. We would like to thank the time and attention of the reviewers as we believe their comments let to a substantial improvement in the quality of our manuscript.

Reviewer remarks:

Reviewer #1: Pereira and colleagues describe a circuit of three nuclei involved in the freezing behaviour after hearing the cessation of sound made by conspecifics. They used a combination of pharmacological and optogenetic silencing together with behavioural experiments. C-fos and tracing data were used to corroborate the results. Overall, the manuscript is short, straight forward and interesting to a wide range of scientists. I have three major concerns that I would ask the authors to address and a number of minor comments that can be easily fixed listed below.

Major concerns:

1. The ArchT expression was not driven by a MGD specific cre-line. So the specificity of the optogenetic silencing was entirely based on the correct (and spatially restricted) injection of the viral vector into the MGD. As the MGD is small and very close to other potential contributors to this circuit like the other parts of the MGB, which can also generate offset responses (Anderson & Linden, 2016), the injection site and specificity needs to be validated. In fact, the results of the silencing look almost too good to be true, which made me wonder if the ArchT actually silenced larger parts of the MGB than just the MGD. This would still be a good, new and interesting result – but would not make claims that might not be justified. Please either show the injection sites of all tested animals to make sure ArchT is expressed only in MGD. Or change the wording to have the whole MGB be part of that circuit, rather than just MGD. Or repeat a couple of the experiments with a cre-dependent ArchT expression in a cre-line that is explicitly expressed in MGD.

The reviewer rightly points out that the specificity of our optogenetic manipulation is entirely based on the spread of the viral infection. It is also dependent on the size of the area reached by the light emitted from the tip of the optical fiber. As we are working with rats, it would have been very difficult to find/generate an MGD specific line, for which it is unclear if specific markers exist.

We thank the referee for pointing out the importance of showing the spread of the infection. We have therefore changed not only the supplementary figure showing the optic fiber placements for MGD but also for LA, such that both now include the infection spread for each animal of the experimental groups. In these figures (S1a Fig, and S3a Fig), the diagrams of coronal sections include the fiber tip placement (colored dots, each animal with a different color) and spread (filled region with same color as dot signaling fiber placement) for all experimental rats (ArchT+Light condition). For control rats we are only showing the placement of fiber tips as in the initially submitted version.

We would like to point out that the MGD of rats is quite big, having ~500um from its dorsal to its ventral border and ~800um from its medial to its lateral border, extending for several millimeters in the anterior-posterior axis. Given that the light is unlikely to activate neurons further than 400um (Gysbrechts et al Journal of Biophotonics 2015), we believe we are mostly inactivating the MGD. We may also have inactivated neurons in the SG and in the dorsal portion of the MGV. SG showed little c-fos expression upon exposure to the sound of movement

with silence gaps, therefore we believe is unlikely to explain the result of our optogenetic inactivation experiment. Even though we did see some neurons in the MGV with c-fos expression, these were mostly in the marginal zone, or shell, of the MG. As these were located mostly in the ventral region of the shell, we believe they are unlikely to have been affected by our manipulation. Still, we have added two sentences to our manuscript, one in the results section and another in the discussion section stating:

Results:

"Furthermore, even though our manipulation affected mostly neurons in MGD, we cannot exclude the possibility that we have silence some neurons in SG and the dorsal region of the MGV (S3 Fig)." (page 6, lines 192-194)

Discussion:

"Particularly relevant to this study is the presence of offset cells in the marginal zone of the MGV, including its dorsal region, which might have been affected by our inactivation experiments (18)."(page 8, lines 254-256)

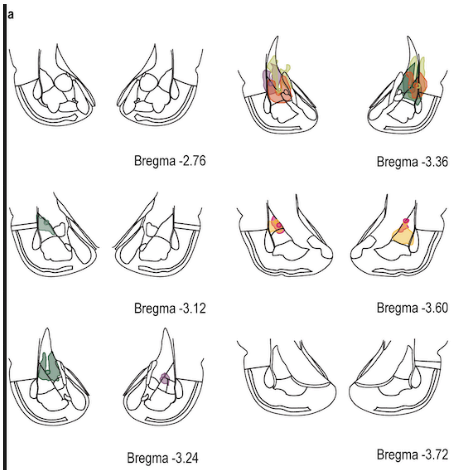

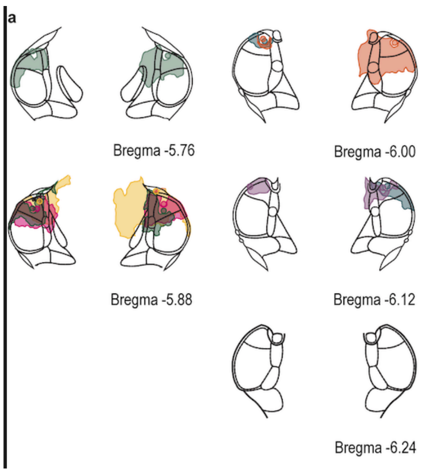

2. What is the spectrum/level of the movement evoked sound? Is it recorded from one or multiple rats? Are you sure there is no vocalization emitted in addition to plain movement sound – need to be tested in ultrasonic range. Please include this information in the methods.

The sound of one single rat was recorded as it moved around in one of the partitions of the social interaction box with bedding on the floor. The sound of this rat moving around was recorded through a microphone, placed over the chamber (Avisoft-UltraSoundGate system 416H, microphones model CM16/CMPA). This allowed recording of ultrasounds. Sections of the recording without vocalizations were chosen for the playback. We had not included this information as we refer to our previous paper, where we had this information. We acknowledge that it is less than ideal to have to look into the methods of a different paper and therefore have now added this information to the revised methods section (page 12, lines 382-387). Please see below the spectrogram of a representative 10s snippet of the sound we used for playback.

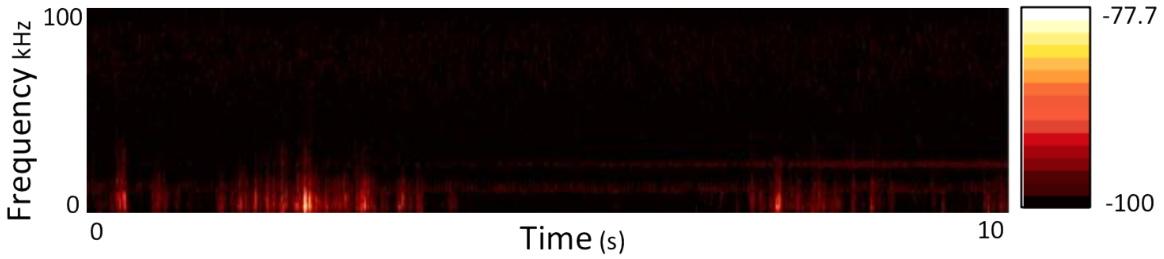

3. How can you be sure that the increase in c-fos expression is really due to exposure to silent gaps? It could just be increased neural activity to any change in stimulus, while the ongoing sound is perceived as "boring" background. Has this been tested with another sound paradigm: e.g. ongoing noise as control (as you have done before), and then some other stimulus where you may have modulated noise instead of silence?

The reviewer suggests that the increase in c-fos expression observed in MGD may be due to a salient change in the auditory stimulus, rather than resulting from the activation of sound offset cells. We agree with the reviewer that this is a possible alternative explanation of the data. Although we could in principle run an experiment as suggested by the referee, we believe the outcome of such an experiment is unlikely to be conclusive. If, for example, we exposed rats to the movement-evoked sound and either increased its intensity, instead of inserting silence gaps, or added another sound, it is very likely that this manipulation would also lead to increases in c-fos expression in multiple MG sub-nuclei including MGD. This scenario is likely given that all MG nuclei, including MGD, show sound onset responses and many cells that are offset cells have both onset and offset responses. If indeed we would find increases in c-fos expression when exposing rats to an additional or louder sound, such finding would not exclude the possibility that the increase in c-fos expression when silence gaps are inserted are at least in part due to activation of offset cells.

Our goal with this experiment was to determine which MG sub-nucleus, that projects to amygdala, was more responsive in our paradigm and target that one for manipulation. Given the literature in rodents and particularly in rats where the response properties of MG neurons were characterized, we believe that a simple explanation for our result is the activation of offset cells which are more prevalent in MGD then in the other sub-nuclei that project directly to the amygdala. Still, to establish that offset cells within the MGD are the ones activated in our paradigm, driving VA and LA to trigger freezing, requires several experiments that we believe would constitute a very interesting follow up study that we are unequipped to do as our lab has switch to work with fruit flies.

We now acknowledge in the revised version of the manuscript the alternative explanation for the c-fos expression proposed by the referee:

"Our findings show that the MGD is the only sub-nucleus of the auditory thalamus projecting directly to LA that is more active when rats are exposed to the sound of movement with silence gaps. Prior electrophysiology studies in rodents report the presence of cells with sound offset responses throughout the MGB with particular prevalence in the MGD (with the highest incidence in the caudal part) and the marginal zones, or shell, of the MGV(17–20). This suggests that *c-fos* positive cells in the MGD may have been activated by the offset of the sound of movement when silence gaps are introduced. In agreement with this hypothesis, we also

observed the presence of robust *c-fos* labelling in the ventral shell of the MGV, in particular in more rostral areas (see S2 Fig and below). Alternatively, c-fos expression may reflect a response to an increase in stimulus saliency resulting from the introduction of silence gaps in the background sound. However, as we failed to see significant changes in the other MG sub-nuclei studied, this alternative explanation would imply that the MDG is the sub-nucleus with strongest sensitivity to changes in saliency. To our knowledge there is no evidence in this direction. Future experiments where activity of MGD neurons is recorded during exposure to sound of movement with either silence gaps or other changes in saliency are necessary to definitively disambiguate between the two possibilities." (page 5, lines 138-154).

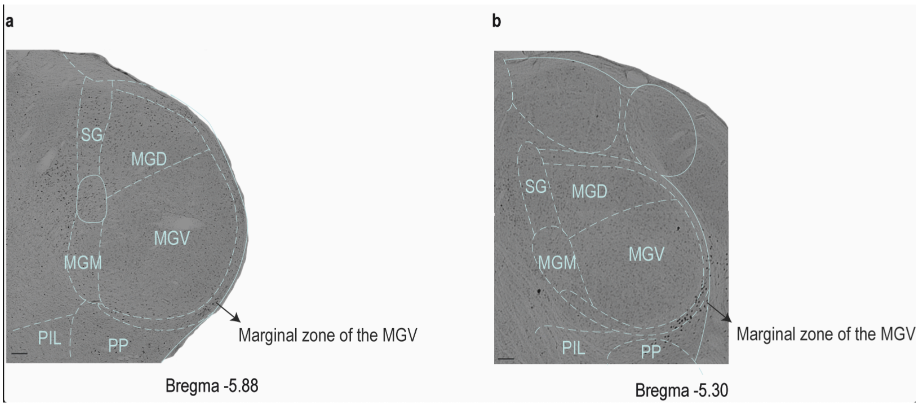

Minor comments:
There are groups which question the validity of the muscimol inactivation of the cortex too, but I would let this one go here.
Line 75: there is a question mark sign – should be an arrow (maybe just say: "White arrow shows...")

We thank the reviewer for having identified this error. It is now corrected.

Fig. 2C: Please provide higher magnification images so that the reader can see neurons rather than what looks like speckles of dirt. A counterstain for e.g. VGLUT or MAP2 would be also very helpful.

The speckles of dirt mentioned by the referee puzzled us for a long time. They are not dirt but neuronal processes, probably of tufted and/or stellate cells very common in the MGB. We have done counter stains with GFPA as at some point we though these might be glial cells and found no overlap. We have added a photomicrograph to supplementary figure 2 showing a higher magnification of the transfected neurons counterstained with NeuN. We have also added a movie (supplemetal movie S1) that shows a higher magnification of transfected neurons that rotate, allowing for a better view of the neuronal processes.

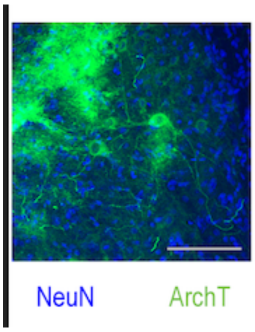

Line 166, 175, 359, 361: typo: kHz

Done

Lines 173/177: ranksum or rank sum – be consistent!

Done

Fig. 4: please indicate in the figure where the PBS or muscimol was injected (e.g. muscimol (into PD)), otherwise the reader needs to scramble though the legend first to get the message of the figure…

We thank the reviewer for this comment and we have now added this information to the figure.

Why were only male mice used? Many journals now insist that both sexes were used, if not a scientific reason prevents the use on both.

Although we do agree with the reviewer that ideally we should have tested both females and males, for us that was not feasible. The current study is part of a larger body of work in our lab devoted to the study of social interactions, which meant working with dyads, duplicating the number of animals required for each data point. Variance in our data was higher as it implicated the behavior of two animals. We worried that also using both genders could increase the variance even further.

To perform the same experiments in female rats, although important and potentially revealing of interesting sex differences, would imply doubling the already large number of animals used. With the intention of following the 3R policy, we decided not to perform comparisons across genders that were not required to address the main point of this study. We work with an outbred strain of rats. Hence, to maintain such a colony was not viable for the large numbers of animals we required, therefore we always imported our animals. This means that we could choose to work with males without leaving 'unused' large numbers of female rats.

Line 259, 349, 351: typo: fiber

Done

Lines 373/374: use dots as decimals

Done

Reviewer #2: Ana et al., reported that an auditory cue, the cessation of movement-evoked sound, induced defensive freezing, and further identified the underlying neural circuits comprising of MGd, ACx and LA. The authors thought this is a unique offset pathway for "cessation of movement-evoked sound" induced defense behavior. The study is mostly based on the behavioral analysis. However, there is major problem in their behavioral design. The authors' interpretation of the behavior results and the conclusion could be completely wrong. The proposed new circuit is the classical circuit for fear conditioning.

1 The defense behavior induced by "cessation of movement-evoked sound" examined in this study was first reported by this research group in 2012 in current biology. This is not an innate behavior (as suggested for impending danger), but learned defense as it required conditioning training with foot shock (Fig. 1a).

The reviewer rightly points out that the freezing behavior triggered by the cessation of movement-evoked sound is not innate but learned. It is not clear to us what the referee means by "as suggested for impending danger". It is our understanding that learned cues can also signal impending danger. In classical fear conditioning tones or other sounds typically predict shock that will occur within seconds of its onset. We have double check the text of our manuscript to make sure that it is clear that we are dealing with a learned cue of threat and added a sentence to explicitly state that this is a learned cue of danger:

"This is a learned cue as it required prior experience with shock" (page 2, line 40)

2 This is more problematic for the interpretation of the whole study. This training can directly establish an association between "cessation of movement-evoked sound" (e.g. caused by the trainee rat) and footshock, i.e. some fear conditioning. That means the nature of the later defense behavior could be simply a learned defense under a context-cue of "cessation of movement-evoked sound". This possibility also explains a similar behavior (in a different context) previously reported by authors in 2012. Unfortunately, this was not examined for these years.

As the reviewer stresses the importance of the mechanisms by which exposure to shock lead to observational freezing. Although, we had not published our work devoted to answering this question, we have now pre-published a separate study on it (doi: https://doi.org/10.1101/800714). Briefly, we found that rats learn to associate their own freezing with shock and thereby freezing by others, detected through the cessation of movement evoked sound, becomes an alarm cue. Whether the freezing-shock association is simply a "cessation of movement evoked sound/shock association" remains unclear. We performed experiments where we masked the silence while exposing observer rats to shock by continuously playing the sound of motion. The next day observer rats still froze in response to freezing by demonstrators. This result suggests that it is not simply sound offset-shock association being learned, but it could also be that we did not mask the silence onset well enough during exposure to shock. Therefore, we could not draw strong conclusions and thus did not include it in our other study.

Even if we had established that silence triggered freezing results from a simple association between the cessation of movement evoked-sound, when one freezes, with shock, and that rats can later use this sound cue as predictive of threat, we believe that it is quite interesting to identify the circuit underlying the detection of this simple learned cue that can be used as a natural social cue of threat.

3 This alternative interpretation explains why the proposed neural circuits for this behavior is in fact the same as that for fear conditioning, including VA, LA, MGB etc. It is not a new circuit.

The reviewer expresses concern that the circuit we identify is the same previously and multiple times implicated in auditory fear conditioning (AFC). In addition, according to the reviewer this is to be expected if the behavior observed results from a simple cessation of movement evoked sound-shock association.

However, to our knowledge, AFC studies focus on the onset of an auditory cue associated with shock. In our case the conditioned stimulus would be the offset of a natural

sound cue. Whether the same circuit we describe is required for learning sound offset-shock association, even when artificial sounds such as pure tones are used, remains to be tested. Still, the circuit we identified is indeed partially overlapping with that of AFC:

1) The lateral amygdala (LA) is clearly involved in both our paradigm and classical AFC.
2) The MGB has been implicated in both AFC to auditory stimuli. Studies using AFC have focused mainly on MGm and PIN, which have been implicated in this form of learning. To our knowledge the role of MGD remained untested, despite its projections to LA. In our study, we report that MGD is important for the expression of freezing upon the cessation of movement sound in rats previously exposed to shocks, but not for freezing triggered by a tone previously paired with shock (the classical AFC paradigm). Therefore, it seems that the MDG is not implicated in freezing triggered by both kinds of threat cue.
3) As for VA, which corresponds to ventral portion of A1 and part of Te3V (Smith et al JCN 2011), its implication in AFC is more complex. Te2/Te3 has been shown to have increased responses to conditioned tones. Although important for remote AFC memory recall it does not seem required for recent AFC memory recall, when pure tones are used. Whether VA (defined by its afferent projection from MGD) is required for recent memory recall of a conditioned tone, remains to be tested. We should point out that in our paradigm whatever was learned during exposure to shock corresponds to a 24hr old memory by the time we tested our rats.

In summary, the extent to which the circuit required for freezing in our paradigm is overlapping with that of AFC needs further investigation. However, the overlap is likely to be partial, as at least according to our results the MDG seems to be differentially implicated in the two paradigms.

However to address the reviewer's concern regarding an overstatement of novelty we have changed the title of our manuscript to read:

"Thamalic, cortical, and amydgala involvement in processing a natural sound cue of danger"

4 The authors should at least establish some correlation between neuronal activity in LA, VA, MGd, with the behavior. Is this really an offset responding pathway? How neurons in LA, VA, MGd respond during the cessation of movement-evoked sound and the following 1 minute silence.

5 Experiments with specific silencing of projections from VA and MGD to LA would be helpful. Is LA activation sufficient for inducing freezing in those trainee rats?

The questions raised by the referee in point 4 and 5 are very good, and we believe would constitute a great follow up study. Our lab has switched from studying rats to fruit flies and therefore we will not pursue this line of experiments further. Still, we felt that identifying key brain regions involved in the response to this natural sound cue of threat (whether learn through classical associative mechanisms or not), implicating MGD in this process (to our knowledge for the first time in any behavior), is a very interesting starting point. Hence the submission in the short format of a report.

Reviewer #3, Jennifer Linden: "A newly identified auditory circuit is involved in processing a natural sound cue of danger"
Pereira AG, Farias M and Moita MA

This article documents the discovery of an auditory circuit that drives freezing following cessation (offset) of movement-evoked rustling sounds, such as would be produced by the freezing of another animal. The circuit involves the dorsal subdivision of the medial geniculate body of the auditory thalamus (MGD), the ventral area of the auditory cortex (VA), and the lateral amygdala (LA). The authors use combined optogenetic and behavioural studies to demonstrate that this circuit is necessary to produce offset-evoked freezing behaviour.

This article is likely to be of wide scientific interest, because it is the first demonstration (to my knowledge) of a neural circuit driving behavioural responses to sound offset (rather than sound onset). Neural responses to sound offsets have been reported throughout the central auditory system, but the perceptual significance of these responses is not yet well understood. This paper provides clear evidence that higher central auditory brain areas contribute to behavioural responses to sound offsets. Moreover, this paper is extremely well-written: systematic and thorough yet succinct. Well done!!

I have a few major comments and several minor suggestions.

MAJOR COMMENTS:

(1) Lines 104-106: "Interestingly, prior electrophysiology studies in anaesthetised rodents showed a higher prevalence of sound offset responses in the MGD relative to other sub-nuclei of the auditory thalamus and higher number of offset cells in the caudal part of MGD." This is potentially misleading. It is true that previous studies have found evidence for offset responses in the MGD, but strong evidence for offset responses has also been reported in the MGV, especially in the "lateral shell" of the MGV. See for example:
He (2001) J Neurosci 21:8672–8679
He (2002) J Neurophysiol 88:2377–2386
Anderson and Linden (2016) J Neurosci 36:1977–1995
The authors should clarify that strong evidence for offset responses has also been reported in MGV, and also explain why this subdivision was not studied here (presumably because it does not project both directly and indirectly to the amygdala?).

We thank the referee for bringing to our attention these relevant papers. Indeed, we did not explore MGV because it does not project to amygdala directly as do the other nuclei. Through visual inspection of the c-fos staining in our experiment, we do observe labeled cells in the marginal zone, or shell, of the MGV, mostly in its anterior ventral portion. We have added a supplementary figure (S2 Fig, see reply to reviewer 1 comment 3) with photomicrographs showing c-fos labeling in the shell of MGV and little or no labeling in the core of this sub-nucleus. In addition, we now cite the suggested papers in the revised version of this manuscript (references number 18, 19 and 20).
Finally we add the following text to the results and discussion section (see also reply to point 1 of referee1):

Results:

"Our findings show that the MGD is the only sub-nucleus of the auditory thalamus projecting directly to LA that is more active when rats are exposed to the sound of movement with silence gaps. Prior electrophysiology studies in rodents report the presence of cells with sound offset responses throughout the MGB with particular prevalence in the MGD (with the highest incidence in the caudal part) and the marginal zones, or shell, of the MGV(17–20). This suggests that *c-fos* positive cells in the MGD may have been activated by the offset of the sound of movement when silence gaps are introduced. In agreement with this hypothesis, we also observed the presence of robust *c-fos* labelling in the ventral shell of the MGV, in particular in more rostral areas (see S2 Fig)." (page 5, lines 138-147).

Discussion:

"Particularly relevant to this study is the presence of offset cells in the marginal zone of the MGV, including its dorsal region, which might have been affected by our inactivation experiments (18)." (page 8, lines 254-256)

(2) Figure 2b: Please comment in the text on the high variance in the MGD data. Is it possible that outliers in the "MGD" cases are at an extreme edge of this subdivision, on the border with other subregions, such as the MGV? See first major comment above.

We thank the reviewer to point out this important aspect. Indeed we do see a wide variation in the number of c-fos positive cells between different animals, however we do not have a clear explanation for the observed variance. For each animal we have slices distributed along the AP axis, and this distribution is similar across animals. Furthermore, for each region (and each AP slice) we took pictures using a specific set of coordinates in relation to anatomic points of reference to guarantee the quantification of cells in the same region of interest of each subdivision. Therefore, we cannot attribute the values of the outliers to different anatomical distributions. We followed the reviewer suggestion and add text to the results and methods sections:

Results:

"We also observe that the average number of *c-fos* labelled cells in the MGD of animals exposed to the sound of movement with silence gaps was quite variable. This variability is unlikely to result from differences in anatomic distribution of *c-fos* positive cells within this sub-nucleus (such as proximity to other regions in the MGB that may also respond to sound offset), since we systematically probed the same region of interest within it (see Methods)." (page 4, lines 112-118)

Methods:

"Sections from comparable anteroposterior levels were selected for scoring and images from each sub-nucleus were taken systematically from the same area based on distance from reference points specific for each anteroposterior level." (page15, lines 469-471)

(3) Figure 2 and associated text: It is quite confusing to use the term "silence" to mean an otherwise continuous sound interrupted by two silent gaps. The stimulus was definitely not "silence". Perhaps other terminology could be used here, e.g. "sound with silent gaps".

We thank the referee for the suggestion, we changed to silence gaps instead of silence whenever applicable.

(4) Figure 3: Comparison between positive results in Fig. 3d ("silence test") and negative results in Fig. 3e ("tone test") is potentially flawed. The "silence test" results show the change in the percentage of time the animals spent freezing during the 1min immediately preceding the cessation of the movement sound and the 1min immediately following cessation. The tone test results show the change in percentage of time the animals spent freezing during the 15sec immediately before the tone and the 15sec of tone presentation. The tone test is likely to be underpowered relative to the silence test, because the time periods used for measuring freezing behaviour were much shorter in the tone test. To address this issue, results for the silence test could be re-computed using 15sec intervals, and either shown or at least mentioned in the text. Also: presumably the laser illumination *timing* must have been different for the silence test and the tone test, with a much longer period of optogenetic activation in the silence test than the tone test? This should also be mentioned as a possible reason for caution about interpretation of the negative result for the tone test. It is possible that the experiment and the analysis were simply less sensitive for the tone test, hence the negative result.

We thank the referee for pointing out the weakness in the comparison of effect of MGD silencing during silence period or the tone, as they have very different durations. As requested by the reviewer we have analyzed freezing during the silence period between the gaps in bins of 15 seconds. We believe this new analysis convincingly shows that the effect during the silence test is not due to a longer inactivation of the MGD. We found that rats in the control group increased freezing to either the tone or the silence within the first 15s, however when we inactivated the MDG, this is only true for the tone.
Freezing change between the baseline (15 sec) and the silence (1st 15 sec):
ArchT+light baseline *vs* ArchT+light silence p= 0.250
Control baseline *vs* Control silence p= 0.008
ArchT+light baseline *vs* ArchT+light tone p= 0.016
Control baseline *vs* Control tone p < 0.001
Together, these results show that 15 sec of silence exposure is enough to trigger a significant increase in freezing in the Control group, but the optogenetic inactivation of the MGD abolishes this increase. However, when the auditory stimulus is a tone, inactivating the MGD has no effect. Therefore, although the tone test may be underpowered due to its shorter duration, we can already appreciate differences in rats' behavior in the beginning of the test stimuli. The differences observed show that the initial response to an aversive auditory stimulus can be abolished by the inactivation of the MGD if the stimulus is the cessation of a sound, but not if it is a pure tone.
Finally, we also performed a within animal comparison of the Δ Freezing (%) in response to either silence or tone again focusing on the first 15s:
ArchT+light silence *vs* ArchT+light tone p = 0.04
Control silence *vs* Control tone p = 0.73
While the two stimuli trigger very similar responses in control animals, the response of animals in the ArchT+light group is different when a tone or silence is presented, supporting the idea that MGD is necessary for freezing triggered by the cessation of sound of motion to silence but not triggered by a conditioned cue.
We added this data to a new supplementary (S4 Fig) and a paragraph in the results section:

"To better compare the role of the MGD in freezing triggered by a conditioned tone or the cessation of movement-evoked sound, we analysed the increase in freezing during the initial 15 seconds of the stimulus (silence gap or tone) in both tests. With this analysis we diminish potential confounds related with differences in the duration of the stimulus and MGD inactivation (60s silence, 75s inactivation *vs* 15s tone and 30s inactivation). We found that after 15s of exposure to either the silence gap or the conditioned stimulus, control animals show a significant increase in freezing (S4 Fig). In contrast, when MGD is inactivated (ArchT+light group), rats increased freezing upon the 15s conditioned tone but not to the first 15s of the silence gap (S4 Fig). Moreover, the same animals (ArchT+light) increase their freezing significantly more during the tone test than during the silence test, despite optogenetic inactivation of the MGD in both cases (S4 Fig). These results show that activity in the MGD is necessary for the display of freezing triggered by the transition from movement-evoked sound to silence but not that triggered by a conditioned pure tone. In line with physiological data, this indicates that the MGD may be preferentially recruited to process the offset of sound(17,18,20). Whether it is the offset cells in MGD that, directly or indirectly, drive activity in LA leading to the expression of freezing, remains to be established. Furthermore, even though our manipulation affected mostly neurons in MGD, we cannot exclude the possibility that we have silence some neurons in SG and the dorsal region of the MGV (S3 Fig). (page 6, 175-194)"

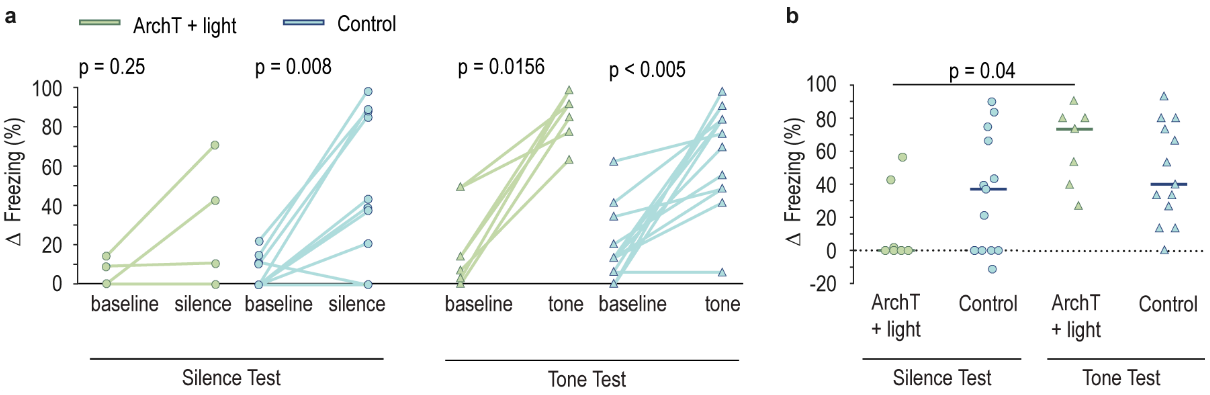

(5) In all of the figures shown, the Control data is pooled from Control-light and Control-ArchT conditions. While it is admirable that the authors tried two different control conditions, the Control-light condition is the far more critical one. Do all results hold when comparisons (e.g. of percent change in freezing) are made between ArchT-light and only Control-light conditions? Supplementary Figures seem to indicate that this is the case, but it would be worth stating in the text for every analysis shown in the main figures. It is especially important to mention whether results held for comparisons to the Control-light condition alone because of the point mentioned in the Methods section, lines 439-442: "Due to the noise generated by the shutter used in the optogenetics experiments, animals that were freezing more than 50% in the 10sec period between the opening of the shutter and silence onset were... excluded". In the tone test (Fig. 3e), the 10sec period between the opening of the shutter and tone cue onset would be 66% of the total pre-tone period (compared to 17% of the pre-silence period in the silence test); was the same exclusion criterion used for the tone test as for the silence test?

We thank the reviewer for addressing these points; we agree it will be informative for the reader to have access to this information.

Regarding the comparisons between the percentage change in freezing (Δ Freezing %) between ArchT-light and the individual controls, all comparisons hold in the case of LA inhibition. Comparing ArchT-light *vs* CT light p=0.0033 and ArchT-light *vs* CT virus p=0.0012. For the experiments where the MGD was inhibited, ArchT-light *vs* CT light p=0.071 and ArchT-light *vs* CT virus p = 0.001. In these experiments we found 2 outliers, one in the ArchT+light group (the value of Δ Freezing (%) of this animal is 78.67 %) and one in the Control light group (the value of freezing during baseline is 20.13%). If we exclude these animals we find that ArchT-light *vs* CT light p=0.038 and ArchT-light *vs* CT virus p = 0.0043. Moreover, if we compare the % of time spent freezing during silence we find that, (and without excluding the outlier) ArchT-light *vs* CT light p=0.042 and ArchT-light *vs* CT virus p = 0.0016. We added to the revised manuscript the following text:

"Similar results were found when analysing the behaviour of animals in the two control groups separately (Wilcoxon Rank Sum test comparing median change in freezing of ArchT+light and Ct light groups, p = 0.003, rank sum = 33; ArchT + light and Ct virus groups, p = 0.001, rank sum = 28 and S1 Fig)." (Page 2-3, lines 66-70).

"When analyzing the behaviour of animals in the two control groups separately the difference between ArchT+Light and Ct virus was again significant, however the difference relative to Ct light failed to reach significance (Wilcoxon Rank Sum test comparing median change in freezing of ArchT + light and Ct light groups, p = 0.07, rank sum = 40; ArchT + light and Ct virus groups, p = 0.017, rank sum = 31) (also see S3 Fig). However, if outliers are removed then all comparisons reveal significant differences across groups (Wilcoxon Rank Sum test comparing median change in freezing of ArchT + light and Ct light groups, p = 0.038, rank sum = 27; ArchT + light and Ct virus groups, p = 0.0043, rank sum = 21)." (Page 5, lines 158-167).

Also, regarding the comment "It is especially important to mention whether results held for comparisons to the Control-light condition alone because of the point mentioned in the Methods section, lines 439-442: "Due to the noise generated by the shutter used in the optogenetics experiments, animals that were freezing more than 50% in the 10sec period between the opening of the shutter and silence onset were excluded".", we believe the reviewer's concern may be related with the fact that the opening of the shutter may have influenced the behavior of the CT light animals in a different way that it influences the behavior of the CT ArchT. However, we would like to clarify that to avoid such confounds, the animals of the CT ArchT group were also subjected to the sound of the shutter – however, the laser was not on. Therefore, the experimental conditions were the same for all 3 groups.

Concerning the exclusion of animals in the tone test that froze more than 50% after the opening of the shutter, we didn't apply the same criterion due to the small sample size – so we used all the animals. There are two animals in the ArchT + light group and two animals in the CT light group that would be excluded based on this criterion. However, if we exclude them we don't see any changes in our results – the median Δ Freezing (%) for the group ArchT + light = 80% (previously 73.33%) and for the group Control = 53.33 % (previously 40%); Wilcoxon Rank Sum test p = 0.1557.

MINOR CORRECTIONS:

line 64: "rats freezing behaviour" -- should be "rats' freezing behaviour"
Done

line 73 (Fig 1 legend): More information should be added here to aid interpretation of the immunohistochemical images: e.g., "Pink, cell bodies labelled with DAPI; green, diffuse LA terminals labelled with ArchT."
Done

line 75 (Fig 1 legend): missing symbol for indicating tip of injector --- arrow?
Done

line 77 (Fig 1 legend): Please clarify here that Control condition includes both Control-light and Control-ArchT conditions. This is mentioned in the text but should also be mentioned in the legend to avoid confusion. See also major comment about mentioning results for Control-light comparison alone
Done

line 147: figure reference should be to Figure 3c and 3d
Done

line 148: figure reference should be to Figure 2c, 2d and 2e
Done

lines 148-149: Anatomical and physiological properties of the MGD in rodents have also been addressed in:
He (2002) J Neurophysiol 88:2377-2386
Zhang, Yu, Liu, Chan and He (2008) Neuroscience 151:293-302
Anderson and Linden (2011) Hear Res 274:48-60
Done

lines 205-208 and lines 210-211 (Fig. 4 legend): More information should be provided here to aid interpretation of the images, e.g. regarding subdivisions of amygdala indicated with dashed lines and localization of anterogradely labelled neurons from MGD or VA.
We thank the referee for mentioning this potentially confusing point, in fact the images in Fig. 4 refer to the MGB (in particular to area MGD) and not to LA. We added information in figure to make it clearer for the reader.

line 259 and elsewhere in methods: "optic fibber" should be "optic fiber"
Done

line 262: "forth day" should be "fourth day"
Done

line 271: "Phosphate Basal Solution" should be "Phosphate Buffered Saline", presumably??
Done

line 275: "bought to Sigma-Aldrich" should be "bought from Sigma-Aldrich"
Done

line 282: "groups were targeted bilaterally" should read "groups received injections targeted bilaterally"
Done

line 312: "to this regions" should be "to these regions"
Done

line 415: "byotinilated" should be "biotinylated"
Done
