# Supplementary material for: Thalamic, cortical, and amygdala involvement in the processing of a natural sound cue of danger
Source: PLoS Biol. 2020 May 12;18(5):e3000674. doi: 10.1371/journal.pbio.3000674 (PMC7217448; doi:10.1371/journal.pbio.3000674)
Supplement: Attachment — Submitted filename: Response to reviewers.docx [file pbio.3000674.s009.docx]

**Response to reviewers**

We are very happy to hear that our revisions to the original manuscript satisfied the reviewers concerns.

Best regards,

The authors
